# N6-methyladenosine modification: A potential regulatory mechanism in spinal cord injury

**DOI:** 10.3389/fncel.2022.989637

**Published:** 2022-09-23

**Authors:** Derong Liu, Baoyou Fan, Jinze Li, Tao Sun, Jun Ma, Xianhu Zhou, Shiqing Feng

**Affiliations:** ^1^Department of Orthopedics, Tianjin Medical University General Hospital, Tianjin, China; ^2^Department of Orthopedics, International Science and Technology Cooperation Base of Spinal Cord Injury, Tianjin Key Laboratory of Spine and Spinal Cord Injury, Tianjin Medical University General Hospital, Tianjin, China; ^3^The Affiliated Hospital of Medical School, Ningbo University, Ningbo, China

**Keywords:** epigenetics, N6-methyladenosine (m6A), post-transcriptional modification, nervous system, spinal cord injury (SCI)

## Abstract

N6-methyladenosine (m6A), an essential post-transcriptional modification in eukaryotes, is closely related to the development of pathological processes in neurological diseases. Notably, spinal cord injury (SCI) is a serious traumatic disease of the central nervous system, with a complex pathological mechanism which is still not completely understood. Recent studies have found that m6A modification levels are changed after SCI, and m6A-related regulators are involved in the changes of the local spinal cord microenvironment after injury. However, research on the role of m6A modification in SCI is still in the early stages. This review discusses the latest progress in the dynamic regulation of m6A modification, including methyltransferases (“writers”), demethylases (“erasers”) and m6A -binding proteins (“readers”). And then analyses the pathological mechanism relationship between m6A and the microenvironment after SCI. The biological processes involved included cell death, axon regeneration, and scar formation, which provides new insight for future research on the role of m6A modification in SCI and the clinical transformation of strategies for promoting recovery of spinal cord function.

## Introduction

N6-methyladenosine (m6A) modification, a type of posttranscriptional modification, has been confirmed to be involved in the post-transcriptional regulation of gene (Roundtree et al., 2017a; Zhao et al., 2017). It was first discovered in mammals in the 1970s (Desrosiers et al., 1974). Notably, m6A is the most common reversible modification found in higher eukaryotic mRNAs (Desrosiers et al., 1974). The dynamic modification of m6A depends on the action of intracellular methylase and demethylase. The former includes methyltransferase-like (METTL) 3, METTL14, Wilms tumor 1-associating protein (WTAP), etc. And the latter includes Fat mass and obesity-associated protein (FTO) and human AlkB homolog 5 (ALKBH5). In addition, m6A-binding proteins also affect RNA metabolism, such as YT521-B homology domain protein family members (YTHDF1-3/YTHDC1-2), heterogeneous nuclear ribonucleoprotein C (HNRNPC) and insulin-like growth factor 2 mRNA-binding proteins 1/2/3 (IGF2BP1/2/3) (Dominissini et al., 2012; Wang et al., 2014; Liu et al., 2015; Huang et al., 2018; Zaccara and Jaffrey, 2020). Moreover, recent studies have demonstrated that m6A is closely related to biological processes of the nervous system, such as brain and cerebellum development, axonal and synaptic formation, gliogenesis, etc (Walters et al., 2017; Yoon et al., 2017; Ma et al., 2018; Xu et al., 2020; Zhao F. et al., 2021).

Spinal cord injury (SCI), a catastrophic condition resulting from a combination of factors, is associated with high rates of disability and fatality and always reduces patient quality of life and imposes a financial burden on families (National SCI Statistical Center [NSCISC], 2016; Ahuja et al., 2017; Tran et al., 2018). Notably, there are no established strategies for completely alleviating SCI and no ideal methods for completely restoring the function of the spinal cord (Venkatesh et al., 2019). Traumatic spinal cord injury is a common type of SCI in clinic (Ahuja et al., 2017). It has two progressive phases: primary injury and secondary injury (Tator, 1995; McDonald and Sadowsky, 2002). The former describes the damage inflicted by direct impact, and the severity of primary injury is proportional to the magnitude of the force applied and the location of the injury (McDonald and Sadowsky, 2002). Secondary injury occurs shortly after primary injury and is accompanied by a series of microenvironmental changes, such as localized hemorrhage and ischemia, inflammation, ionic and neural factor imbalance, glial scarring, and programmed cell death (PCD) (McDonald and Sadowsky, 2002; Fan et al., 2018). Therefore, reducing secondary injury and enhancing functional recovery are key for treating SCI. Fully elucidating the pathogenic mechanisms of SCI is especially critical. Recent studies have found that after SCI, the overall m6A level in the lesion site is increased, and the content of related regulatory factors, such as METTL3 and METTL14, are increased (Xing et al., 2021; Wang et al., 2021; Gao et al., 2022). Furthermore, it was discovered that the specific knockout of mettl14 helps functional recovery after SCI and reduces neuronal apoptosis (Wang et al., 2021; Gao et al., 2022). However, the function of m6A modification in SCI has yet to be fully elucidated. The pathological changes in nerve-related cells and repair processes after SCI may be related to RNA m6A modification, and determining how m6A modification influences these changes may provide insights into novel therapeutic strategies for SCI.

In this review, we summarize the current state of research on m6A modification and emphasize the regulatory mechanism of this type of modification in various pathological processes associated with dysfunction of the nervous system after injury and subsequent tissue repair after SCI to provide a theoretical basis for future research on SCI.

## The regulatory mechanism of N6-methyladenosine modification

Since the discovery of m6A modification, researchers have continued to explore its mechanism and function. With the emergence of various sequencing technologies, such as m6A-seq, MeRIP-seq, m6A-CLIP, and m6A-sensitive HRM analysis, etc., it has been found that m6A modification is ubiquitous in coding and non-coding RNAs (Dominissini et al., 2012; Coker et al., 2019; Wang and Jia, 2020). The deposition of m6A on RNA affects mRNA metabolism, including mRNA nuclear export, splicing, translation, transcription, and degradation (Roundtree et al., 2017b; Huang et al., 2018; Liu J. et al., 2020; Cho et al., 2021; Mendel et al., 2021). Interestingly, numerous studies have confirmed that m6A modification sites are conserved in mRNA and that m6A preferentially binds to regions near stop codons or 3′ and 5′ untranslated regions (Meyer et al., 2012; Meyer et al., 2015). Notably, the conserved mRNA sequence to which m6A binds is generally “RRACH,” where R represents adenine or guanine and H can represent adenine, cytosine, or uracil (Harper et al., 1990). Moreover, successful methylation of the sixth N of adenylate is inextricably linked to m6A-regulating factors, including “writers,” “erasers,” and “readers” (Zaccara et al., 2019).

### Writers

Intracellular RNA methylation often requires co-catalysis by various enzymes, which are named “writers” (Oerum et al., 2021). The methyltransferase complex, which consists of a heterodimeric core formed by METTL3-METTL14 and additional enzymes, such as WTAP (Liu et al., 2014; Ping et al., 2014), normally catalyzes m6A modification (Liu et al., 2014). METTL3, which has been widely studied since it was first discovered in 1997, is known to be the catalytic core of the methylase complex (Bokar et al., 1997; Oerum et al., 2021). Another enzyme, METTL14, plays a synergistic role with METTL3, as both are essential components of the methylase complex (Wang et al., 2016). Binding of METTL14 to RNA enhances the methylase activity of METTL3 and stabilizes the complex structure (Wang et al., 2016).

In addition to Mettl3/14, the role of other writers is also worth exploring. First, WTAP plays a regulatory role in the methylase complex, linking the complex to RNA, and deletion of WTAP results in in aberrant gene expression and alternative splicing (Ping et al., 2014). Recent research on the development and progression of ataxia and neuronal degeneration has revealed that WTAP expression is associated with disease progression and prognosis (Yang et al., 2022). WTAP-deficient mice not only had lower methylation levels in cerebellar Purkinje cells, but they also developed cerebellar atrophy and ataxia over time (Yang et al., 2022). Moreover, METTL16, another member of the METTL family, binds to U6 snRNA, ncRNAs, lncRNAs, and pre-mRNAs to catalyze methyl synthesis and is implicated in RNA splicing and translating (Pendleton et al., 2017; Warda et al., 2017; Satterwhite and Mansfield, 2022). Additionally, METTL16 can promote translation initiation by interacting with eukaryotic initiation factor 3a/b and rRNA in the cytoplasmic matrix, which is dependent on Mtase domain of METTL16 (Su et al., 2022). Furthermore, translation-related rRNAs can be methylated by another methylase, METTL5. METTL5 is essential for cell activity and differentiation potential and is required for effective translation (Ignatova et al., 2020). Mettl5 deficiency reduces overall translation rate, cell pluripotency, and differentiation potential in mouse embryonic stem cells (Ignatova et al., 2020). Additionally, cell translation and proliferation are related to ZCCHC4, a novel m6A writer that can interact with human 28S rRNA and mRNAs *in vitro* and *in vivo* (Ma et al., 2019). A study shows that ZCCHC4 knockout eliminates m6A modification in 28S rRNA, reduces global translation, and inhibits cell proliferation (Ma et al., 2019).

### Erasers

Demethylases can remove methyl groups from nucleotides, and the discovery of m6A demethylases, generally known as “erasers,” reveals that the m6A modification of RNA may be reversed dynamically (Yu et al., 2018). FTO and ALKBH5, both of which are AlkB proteins, can effectively decreased m6A levels (Jia et al., 2011; Zheng et al., 2013). FTO was the first demethylase to be discovered (Jia et al., 2011). Guifang Jia identified the enzyme “FTO” as m6A demethylase in 2011 and established that m6A is the predominant FTO substrate in the nucleus *in vivo* and *in vitro* (Jia et al., 2011). In addition to fat metabolism, FTO has recently been shown to be involved in nervous system pathologies in different contexts (Fischer et al., 2009; Li et al., 2017; Walters et al., 2017; Zhuang et al., 2019).

AlkB homolog 5, another enzyme capable of reversing m6A modification, has also been implicated in posttranscriptional RNA regulation, including mRNA splicing, stability, export and RNA metabolism (Zheng et al., 2013; Covelo-Molares et al., 2021). Inactivation of ALKBH5 causes an increase in m6A levels on mRNAs, and studies have shown that ALKBH5 is essential for the progression of non-neoplastic and neoplastic diseases of the reproductive, immune, circulatory, and nervous systems (Zheng et al., 2013; Cheng et al., 2021; Dong et al., 2021).

### Readers

Eukaryotes produce a variety of proteins that can bind to the m6A modification site and affect RNA translation, splicing, and disintegration and other biological processes (Shi et al., 2017). These proteins are referred to as “readers” and include, most notably, YTH domain family protein 1/2/3(YTHDF1/2/3), YTH domain containing 1/2(YTHDC1/2), HNRNPC, and IGF2BP1/2/3 (Dominissini et al., 2012; Wang et al., 2014; Liu et al., 2015; Huang et al., 2018; Zaccara and Jaffrey, 2020).

YTHDF2 interacts with the m6A modification site on RNA, increasing the likelihood of RNA degradation (Wang et al., 2014). YTHDF2 exerts its effect through several pathways. For instance, YTHDF2 accelerates RNA degradation by recruiting the CCR4/NOT complex (Du et al., 2016). It was also shown that YTHDF2 regulates m6A-mediated RNA decay through the YTHDF2-HRSP12-RNase P/MRP axis (Park et al., 2019). Additionally, after YTHDF1 binds to m6A-tagged mRNAs in the cytoplasm, it stimulates ribosome occupancy of its target mRNA and acts in concert with initiation factors to improve the efficiency of mRNA translation (Wang et al., 2015). YTHDF3, another m6A binder, has been found to have two functions (Shi et al., 2017). It can work with YTHDF1 and YTHDF2 to increase mRNA translation or speed up methylated mRNA degradation, respectively (Shi et al., 2017). Furthermore, YTHDC1, a particular nuclear ribonucleic acid-binding protein, promotes alternative splicing by attracting the RNA splicing factor SRSF3 and preventing SRSF10 from binding to mRNAs in the nucleus (Xiao et al., 2016). It also regulates mRNA export from the nucleus to the cytoplasm (Roundtree et al., 2017b). Another member of this family, YTHDC2, is capable of altering the translation efficiency and mRNA abundance of its targets (Hsu et al., 2017). In addition, HNRNPC is also a common nuclear protein that detects and binds to m6A-modified sequences in mRNAs and lncRNAs, affecting target RNA abundance and splicing (Liu et al., 2015). In contrast to YTHDF2, IGF2BP1/2/3 are novel m6A readers that can protect m6A-modified mRNAs from degradation (Huang et al., 2018). They help thousands of potential mRNA targets remain stable and undergo translation (Huang et al., 2018). Recently, a novel m6A “reader,” Prrrc2a, which is strongly associated with oligodendrocyte formation and axonal myelination, was identified by Wu R. et al. (2019). Their study found that Prrc2a can stabilize Oligo2 mRNA after binding to the m6A site (Wu R. et al., 2019). Additionally, when Prrc2a was removed, mice showed developmental abnormalities, such as enlarged lateral ventricles and significantly reduced myelin sheaths (Wu R. et al., 2019).

## N6-methyladenosine modification after spinal cord injury

The nervous system is a multicellular network, and the close interactions among numerous nerve cells, such as neurons and glial cells, is essential for the coordination of its functions (Sousa et al., 2017). Direct damage to the spinal cord can disrupt the blood–spinal cord barrier and cause local blood supply insufficiency, directly resulting in cell death (Ahuja et al., 2017). Notably, the subsequent changes in the internal environment of the spinal cord broaden the scope of injury, and local structures undergo corresponding changes, including scar formation and axonal regeneration (Hara et al., 2017; Fan et al., 2018). M6A modifications are at higher levels in the nervous system (Meyer et al., 2012). Changes in M6A content and associated regulatory factors influence nervous system development and function. For instance, METTLl14 deficiency reduced m6A levels in mouse cerebral cortex and prolonged cortical neurogenesis (Yoon et al., 2017). A study has also demonstrated that the deletion of the methylase METTL3 results in ataxia, hypoplastic development of the mouse cerebellum, and an increase in the apoptosis of immature granulosa cells (Wang et al., 2018). Another study found that peripheral nerve damage raised the levels of FTO, G9a protein, and decreased Ehmt2 mRNA m6A methylation level, all of which contributed to the development of neuropathic pain. Additionally, it was shown that reducing FTO expression in the dorsal root ganglion can reduce neuropathic pain caused by injury (Li et al., 2020).

Recent studies have also reported that after SCI, the levels of m6A as well as writers, such as mettl3 and mettl14, in tissues rise dramatically and specific knockout of methylase can alleviate the severity of SCI (Wang et al., 2021; Xing et al., 2021; Gao et al., 2022). This indicates that dynamic m6A modification has a strong potential to regulate the injury mechanism after SCI and influencing functional recovery.

### N6-methyladenosine modification and cell death after spinal cord injury

The structural and functional integrity of the spinal cord are the foundations for proper physiological activity (Ahuja et al., 2017). However, SCI is a multistep disorder usually accompanied by massive neuronal cell death, which is one of the reasons why SCI is difficult to treat (Anjum et al., 2020). In addition to the cell destruction induced by direct impact, secondary injury changes the internal environment and structure of the spinal cord and induces PCD of nerve cells (Fan et al., 2018; Shi et al., 2021). Therefore, preserving nerve cells and reducing or even eliminating cell death are critical for the treatment of SCI. To achieve better treatment outcomes, it is essential to explore the mechanism of PCD after SCI.

Programmed cell death is tightly linked to m6A modification (Wang et al., 2020; Lan et al., 2021; Shen et al., 2021; Liu et al., 2022). Apoptosis is a common form of PCD in the nervous system (Fricker et al., 2018). A study showed that knockout of mettl3 results in massive apoptosis of newborn cerebellar granule cells, resulting in dysplasia in the mouse cerebellum (Wang et al., 2018). Similarly, Mettl3 deficiency in the mouse hippocampus increases local apoptosis and alter the cell cycle (Zhao F. et al., 2021). In addition, after ischemic brain injury, overexpression of YTHDC1 reduces neuronal apoptosis (Zhang et al., 2020).

**FIGURE 1:**
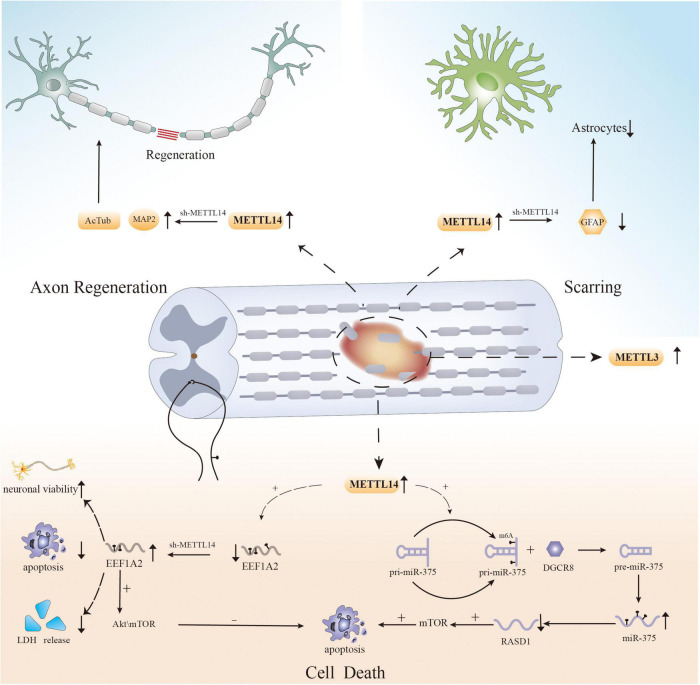
The role of m6A in spinal cord injury.

Recently, several studies have shown that methylation regulators can influence cell survival after SCI by regulating m6A levels (Figure 1). Haoyu Wang et al. verified that significant neuronal death and cell dysfunction occur at the site of injury in a rat spinal cord contusion model (Wang et al., 2021). Moreover, m6A levels were increased, and the expression of the “writer” mettl14 is increased (Wang et al., 2021). Surprisingly, inhibiting local Mettl14 expression lowers overall m6A levels and the severity of SCI in experimental animals while also promoting motor function recovery after injury (Wang et al., 2021). To explore the changes at the cellular level, the researchers performed HE staining and immunofluorescence (Wang et al., 2021). The results showed the presence of fewer reactive astrocytes in the injury area and more surviving neurons in the mettl14 knockout group compared to the control group (Wang et al., 2021). More importantly, further experiments also showed that overexpression of Mettl14 can induce apoptosis *in vitro*, as Mettl14 can promote the conversion of pri-miR-375 to miR-375, which is related to apoptosis and inhibits neural recovery (Wang et al., 2021). In addition, increased expression of METTL14 during SCI mediates the m6A modification of EEF1A2, which accelerates neuronal degeneration through the apoptotic pathway and impairs recovery after injury (Gao et al., 2022). EEF1A2 expression is reduced after SCI, while silencing of mettl14 increases EEF1A2 levels, decreases inflammatory cytokine production, and reduces neuronal degeneration in the spinal cord (Gao et al., 2022).

The above experiments show that the regulation of cell death after SCI, particularly neuronal apoptosis, is influenced by RNA m6A modification, providing a new direction for reducing cellular dysfunction and promoting functional recovery. However, apoptosis is not the only cause of cell loss after injury, and previous studies have shown that other forms of PCD, such as ferroptosis, autophagy, and necroptosis, also mediate cell death after SCI (Fan et al., 2016; Zhou et al., 2020; Feng et al., 2021; Shi et al., 2021). There have been multiple studies on the effect of m6A modification on PCD in different disorders (Yang et al., 2019; Lan et al., 2021; Shen et al., 2021); however, there has been no research on the relationship between m6A modification and other forms of PCD after SCI. Therefore, to properly elucidate the pathogenic mechanism of SCI, researchers must examine the role of m6A modification in other types of PCD after SCI.

### N6-methyladenosine modification and axonal regeneration after spinal cord injury

Another cause of functional deficiency following SCI is the disruption of spinal nerve continuity (Ramer et al., 2014; Tran et al., 2018; Varadarajan et al., 2022). Unfortunately, compared to that of the peripheral nervous system, the axonal regeneration capacity of the central nervous system is extremely limited (Hutson and Di Giovanni, 2019; Avraham et al., 2021). Failure of regeneration results in permanent loss of neurological function. Although we have conducted in-depth research on the internal and external environment and regeneration mechanisms after axonal injury, complete axonal regeneration is difficult to achieve (Liu et al., 2011; Varadarajan et al., 2022). Studies have shown that changes related to gene expression can effectively regulate axonal regeneration, which involves physiological processes such as translation and transcription (Moore et al., 2009; Song et al., 2015; Mahar and Cavalli, 2018). Recent research on m6A modification also revealed that RNA modification can influence axonal regeneration, providing a solid theoretical basis for our ongoing research on axonal regeneration (Weng et al., 2018; Zhang et al., 2021; Qi et al., 2022).

In the nervous system, regeneration of neuronal axons is likewise affected by m6A modification. Following sciatic nerve damage, the levels of m6A-tagged transcripts associated with axonal regeneration are increased in mouse dorsal root neurons, facilitating axonal regeneration. Primary neurite length is considerably decreased *in vitro* when METTL14 is knocked out, as is the capacity to increase the axon length *in vivo*. In addition to that in the peripheral nervous system, Pten deletion-induced axonal regeneration in CNS neurons is considerably impeded following METTL14 loss. Furthermore, the YTHDF1 reader is required for injury-induced protein translation and axonal regeneration in neurons (Weng et al., 2018). Additionally, another study pointed out that FTO can reduce RNA m6A levels in axons and dynamically regulate local protein translation (Yu et al., 2018). After inhibition of intraneuronal axonal FTO expression by rhein, m6A levels are significantly decreased, and axonal elongation is inhibited (Yu et al., 2018). Interestingly, Mengru Zhuang’s team discovered that the m6A-binding protein YTHDF1 recognizes transcripts and regulates the translation of Robo3.1, which is modified by m6A, provides axonal pathfinding guiding signals, and affects the guidance of crossing axons of spinal cord commissure neurons (Zhuang et al., 2019). In addition, YTHDF1 and YTHDF2 are highly expressed in cerebellar granule cell axons *in vitro* and *in vivo*, and knock out of these proteins might enhance axonal development (Yu et al., 2021). To govern neuronal axonal development, YTHDF1 and YTHDF2 synergistically regulate Wnt5a signaling, which is involved in axonal guidance and can influence axonal development (Yu et al., 2021).

Recently, m6A modification was shown to have the potential to regulate axonal regeneration after SCI (Figure 1). In an experiment on SCI in zebrafish and mice, MeRIP-seq and RNA-seq analysis of injured tissue after SCI revealed that RNAs that showed obvious differences in m6A levels, such as hsp90ab1, taf1, igf2bp1, and tp53, were associated with axonal growth and neuronal development (Xing et al., 2021). Simultaneously, the expression of METTL3 was found to be upregulated in local tissues in mouse and zebrafish SCI models, as well as in neural stem cell and astrocyte SCI models (Xing et al., 2021). This is the first study on the role of RNA m6A modification in SCI, and the results suggest that dynamic changes in the methylation of associated genes have an effect on axonal regeneration (Xing et al., 2021). In addition, specific knockout of METTL14 can significantly increase the expression of AcTub and MAP2 after SCI, which are two markers associated with axons whose expression is decreased after SCI. These findings indicate that METTL14 is involved in the regulation of axons after SCI (Wang et al., 2021). And another study found that METTL14 catalyzes the m6A methylation of EEF1A2 mRNA (Gao et al., 2022). Knockdown of mettl14 can increase the level of EEF1A2, and the opposite occurs after mettl14 overexpression (Gao et al., 2022). Moreover, the reduction in EEF1A2 expression after SCI inhibits the Akt/mTOR pathway, which previous studies have shown to affect pathway regeneration (Zhao Y. et al., 2021; Gao et al., 2022). Therefore, m6A modification may have an effect on nerve recovery. The results of the abovementioned experiments suggest that m6A modification could be a potential strategy for affecting axonal regeneration after SCI.

### N6-methyladenosine modification and scarring after spinal cord injury

One of the secondary characteristics of SCI is the aggregation of a considerable number of reactive astrocytes, which always results in localized scarring (Hara et al., 2017). Spatially, scars can be used to isolate damaged tissue and prevent damage from spreading further (Tran et al., 2018). In addition to exerting a protective effect, scars inhibit nerve regeneration, which is closely related to the recovery of spinal cord function (Silver and Miller, 2004). Recently, research has shown that scar formation after injury does not necessarily hinder axonal regeneration but may actually promote recovery (Anderson et al., 2016). Compared to that of astrocytes, the role of pericytes in scar formation has received less attention. Pericytes are also crucial for the scarring process (Göritz et al., 2011; Dias et al., 2018). Therefore, research on scar formation from the perspective of m6A modification could open up a new field of research related to SCI.

N6-methyladenosine modification can regulate the physiological functions of astrocytes (Huang et al., 2020; Teng et al., 2021). In a study on major depressive disorder, it was verified that circSTAG1 can bind to the demethylase ALKBH5 in the mouse hippocampus, decreasing ALKBH5 levels to alter the m6A level of FAAH mRNA and limit FAAH expression (Huang et al., 2020). Ultimately, astrocyte dysfunction and astrocyte loss are reduced (Huang et al., 2020). Additionally, METTL14 knockdown reduces m6A levels in the substantia nigra, decreases TH expression, and enhances microglial and astrocyte survival (Teng et al., 2021).

Recently, several studies have shown that changes in m6A modification affect the aggregation of astrocytes following SCI (Figure 1; Wanner et al., 2013). Lingyan Xing et al. found that the expression of METTL3 in astrocytes increases dramatically after SCI, possibly affecting the activation and proliferation of cells (Xing et al., 2021). Although more research is needed, the results indicate a new direction for the study of astrocytes after SCI. Moreover, another study reported that GFAP expression was decreased and the number of astrocytes produced at the injury site was reduced in an SCI model with selective deletion of Mettl14 compared to the control group (Wang et al., 2021). Surprisingly, *in vitro*, lack of Mettl14 was shown to reduce the apoptosis of C8-D1A murine astrocytes after simulation of SCI-induced apoptosis with H2O2 (Wang et al., 2021). This implies that m6A modification is linked to astrocyte survival after SCI, which can alter scar formation. However, since there are only few related studies, the relationship between m6A and astrocytes after SCI still needs to be further explored.

While astrocytes are involved in scarring postinjury, the role of pericytes in SCI cannot be ignored (Dias et al., 2018). Pericytes are involved in the establishment of the blood–brain barrier and the blood–spinal cord barrier, as well as the stability of the internal environment of the brain and spinal cord (Cheng et al., 2018; Sweeney et al., 2019). Previous studies have shown that pericytes are closely related to the formation of scars and the recovery of function after SCI (Dias et al., 2018; Hesp et al., 2018; Zhu et al., 2022). Some studies have confirmed that m6A modification in pericytes is involved in the occurrence and development of hypertension and diabetes (Wu Q. et al., 2019; Suo et al., 2022). For instance, Qingbin Wu et al. discovered that in pericytes, mRNAs undergo m6A modification in coding regions under hypertensive conditions. Subsequent GO and KEGG enrichment analyses revealed that the differentially expressed genes are linked to hypertension genes and pathways. This suggests that changes in m6A modification in pericytes play a role in the pathogenesis of vascular diseases such as hypertension (Wu Q. et al., 2019). Moreover, a recent study found that diabetes-induced pericyte dysfunction is associated with changes in RNA m6A levels, which are regulated by m6A-related enzymes and proteins (Suo et al., 2022). Selective METTL3 silencing can reduce YTHDF2-induced degradation of PKC, FAT4, and PDGFRA mRNA, reducing the occurrence of diabetes-induced vascular complications and pericyte dysfunction (Suo et al., 2022).

## Future directions related to the role of N6-methyladenosine modification after spinal cord injury

**FIGURE 2:**
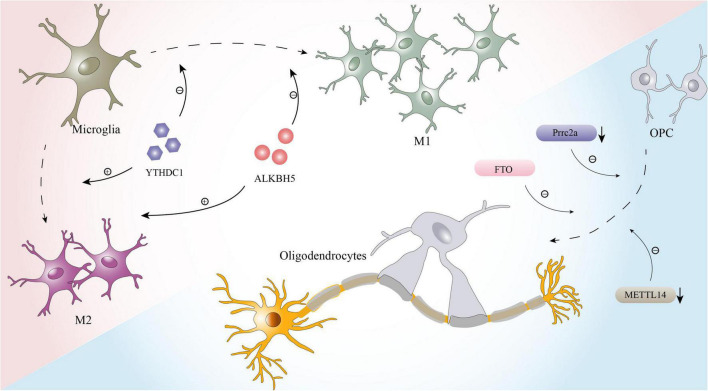
The effects of m6A on microglia and oligodendrocytes.

In addition to the pathological processes mentioned above, the effects of local inflammation and myelination dysfunction on prognosis after SCI should not be ignored (Plemel et al., 2014; Zrzavy et al., 2021), and m6A modification is also likely to be involved in these effects. Microglia, which are key factors affecting inflammation after SCI, have two polarization states, the proinflammatory M1 phenotype and the anti-inflammatory M2 phenotype (Lan et al., 2017). After injuries such as stroke, cerebral hemorrhage, SCI, M1 polarization of microglia is often induced (Fan et al., 2018; Liao et al., 2020; Sun et al., 2020). While M1 microglia play a defensive role, they also aggravate neuroinflammation and nerve cell damage, affecting the recovery of nervous system function (Fan et al., 2018). Therefore, reducing the polarization of M1 glial cells or driving their conversion to the anti-inflammatory M2 phenotype can aid nerve recovery and lessen secondary damage (Liu W. et al., 2020). According to recent studies, m6A modification plays a critical role in glial phagocytosis and polarization (Figure 2; Li et al., 2021; Zhou et al., 2021; Chen et al., 2022). A study on uveitis found that deletion of the m6A reader YTHDC1 enhances the M1 polarization of microglia and accelerates inflammation (Zhou et al., 2021). Furthermore, another bioinformatics study showed that m6A has a high potential to modulate the microglia-mediated inflammatory response. A large number of m6A-modified transcripts are among the genes that are differentially expressed between different subtypes of microglia (Li et al., 2021). Researchers have also observed that when microglia are active, m6A levels of the transcripts of many pro- and anti-inflammatory components are altered (Li et al., 2021).

Furthermore, oligodendrocytes, whose primary function is to form the myelin sheath of axons and contribute to the efficient and rapid transmission of information, are inextricably linked to myelin regeneration during the process of nerve repair after SCI (Bradl and Lassmann, 2010; Sankavaram et al., 2019). In recent years, it was proven that m6A modification plays a key role in the development and maturation of oligodendrocytes and maintains the normal function of oligodendrocytes (Figure 2; Wu R. et al., 2019; Xu et al., 2020). For example, Prrrc2a, a novel m6A “reader” identified by Wu R. et al. (2019) is strongly associated with oligodendrocyte formation and axonal myelination. When prrc2a is specifically knocked out, the proliferation and differentiation of OPCs are affected, and the number of mature oligodendrocytes is markedly reduced (Wu R. et al., 2019). Moreover, axons in the corpus callosum exhibit hypomyelination (Wu R. et al., 2019). Interestingly, Xu et al. (2020) performed RNA-seq and m6A-seq of OPCs and successfully induced the differentiation of OPCs from neonatal mice into oligodendrocytes. When METTL14 is inactivated by Cre-loxP, the number of mature oligodendrocytes in postnatal mice is significantly reduced, but the formation and proliferation of OPCs are not affected (Xu et al., 2020).

## Conclusion

This review discusses in detail the current status of research on m6A modification and the relationship between m6A modification and pathophysiological processes after SCI, including cell death, axonal regeneration, and scarring. Although there has been research on the role of m6A modification in some neurological diseases, such as Alzheimer’s disease and stroke, research on the role of this posttranslational modification in SCI is still in its infancy. Research on this topic is limited to bioinformatics analysis of gene expression and differential expression at the tissue and cell levels, and studies on the specific mechanism of m6A modification after SCI are extremely rare. Simultaneously, the only m6A modification-regulating molecules that have been studied after SCI are “writers,” and more research on the impact of demethylases and binding proteins after SCI is needed. The importance of m6A modification in neurological diseases cannot be overstated. This dynamic modification could be a possible target for influencing the pathological process of SCI and promoting recovery of spinal cord function. Clearly, the role of m6A modification in SCI needs to be explored further.

## Author contributions

DL, BF, and JL contributed the central idea and wrote the manuscript. JM and TS collected the related data. XZ and SF participated in key revisions of the manuscript and finalized the final version. All authors contributed to the revision of the manuscript and approved the submitted version.
